# Maize productivity and soil properties in the Loess Plateau in response to ridge-furrow cultivation with polyethylene and straw mulch

**DOI:** 10.1038/s41598-019-39637-w

**Published:** 2019-02-28

**Authors:** Hao-Liang Deng, You-Cai Xiong, Heng-Jia Zhang, Fu-Qiang Li, Hong Zhou, Yu-Cai Wang, Zhan-Rui Deng

**Affiliations:** 1 College of Water Conservancy and Hydropower Engineering, Gansu Agricultural University, Lanzhou, 730070 China; 2 Northwest Institute of Eco-Environment and Resources Chinese Academy of Sciences, Lanzhou, 730000 China; 3 The Institute of Arid Agroecology, School of Life Sciences, Lanzhou University, State Key Laboratory of Grassland Agroecology, Lanzhou, 730000 China; 4 College of Food science and Engineering, Gansu Agricultural University, Lanzhou, 730070 China

## Abstract

Ridge-furrow with full film mulching (RFFM) is widely used in the Loess Plateau (LP) to increase maize yield. However, continuous RFFM application may cause excessive depletion of soil organic carbon (SOC) and soil water storage (SWS). The present study tested four production systems, namely, (1) RFFM; (2) ridge-furrow with polyethylene film and straw mulching (RFFSM); (3) non-contoured seedbed with film mulching (FFM); and (4) non-contoured seedbed without mulching (CK) in 2013 and 2014 to identify an optimal technique to increase maize yield yet minimizing the negative effects. SWS under RFFSM was significantly higher by 5.4% and 13.4% compared to RFFM and CK, respectively. The changes in SOC were −0.2, −0.2, and −0.4 g·kg^−1^ for RFFM, FFM, and CK, respectively, and 0.3 g·kg^−1^ for RFFSM. Increased root residue and extra external carbon input to soil under RFFSM directly contributed to SOC recovery. RFFSM had a comparable grain yield but higher water use efficiency compared to RFFM. The combination of RFFSM is promising for improving SOC stocks, water storage, and maize productivity.

## Introduction

The semiarid Loess Plateau (LP) of China is characterized by low, erratic rainfall, and high evaporation. The LP is also subjected to severe soil erosion and land degradation. Smallholder-based rain-fed farming is the principal form of crop production in the LP^1^. Low and variable agricultural productivity has been a major challenge to the continuously increasing population size and poverty rate in most areas of the LP. These issues result from both natural obstacles and technical restrictions. First, the natural rainfall pattern is incapable of supplying water at critical crop growth stages. More than 60% of the annual precipitation occurs between July and September, while most spring crops are sown between March and April^2^. The temperature in early spring is also generally too low to ensure optimal seedling establishment^3^. In addition, precipitation during the growing season occurs mainly as light rain showers or rainstorms. Traditional farming techniques have a low capacity for rainwater collection, and these techniques often result in heavy surface runoff and soil degradation. Spring maize is a major crop in the LP and is grown on 27.3% of the total agricultural area^4^. However, limited water availability and low soil temperatures at the maize seedling stage often lead to low yield.

Given the local climate and topographic characteristics in the LP, in-field rainwater collection technologies incorporating evaporation reduction measures may improve soil water retention and increase crop productivity. Many attempts have been made to increase the efficiency and suitability of *in situ* rainwater harvesting and mulching technologies. For example, the transformation of sloped farmland to terraces has been used to increase soil water conservation and reduce surface runoff and soil erosion in hilly areas of the LP. During the rainy season, the soil water content in the terraces has greater temporal stability^5^. The subgroove water harvesting technique with a 40-cm depth can increase rainwater-saving capacity and crop yield in the LP^6^. In addition, an optimal distribution of alternating ridges and furrows has great potential for intercepting runoff and harvesting rainfall at the crop field scale, especially on sloping land^7^. Mulching studies, including material selection and mulch patterns, have shown promising results. For example, in semiarid regions where gravel and nearby sand deposits are readily available^8,9^, mulching soils with gravel sand is an effective method for increasing storage soil water and limiting evaporation in fruit and vegetable fields^10^. Crop straw is widely used to improve crop productivity by reducing evaporation and enriching the soil carbon pool in areas where local animal husbandry does not compete for this crop stubble^11^.

Dryland maize cultivation in the LP has evolved from a traditional non-contoured seedbed into film-mulched flat seedbed and the current, widely used ridge-furrow film-mulching (RFFM) technique^12,13^. RFFM is a farming technique that enhances local food security in the LP. The RFFM can increase the penetration of light rain into the soil and reduce surface runoff during heavy rains when ridges are built along the contour. It can also improve soil water content by preventing water evaporation and prolonging the period during which moisture is available to the crop^10,14^. However, some negative soil effects can result from long-term continuous application of RFFM. These include poor infiltration of late season rains and soil carbon pool loss due to accelerated SOC decomposition^15,16^. The effectiveness of RFFM could be increased by implementing appropriate modifications. In this study, we used a local maize (*Zea mays* L.) hybrid, JK-3, and evaluated four different production systems at a typical semiarid LP site. Our objectives were to: 1) determine the overall effectiveness of an updated RFFM on soil water storage (SWS), thermal modification, crop growth, grain yield, and water use efficiency in two growing seasons, and 2) compare the differences in SWS, soil nutrients, microbial activities, and grain yield between the updated RFFM and other alternative practices.

## Results

### Spatiotemporal dynamics of SWS

**Figure 1:**
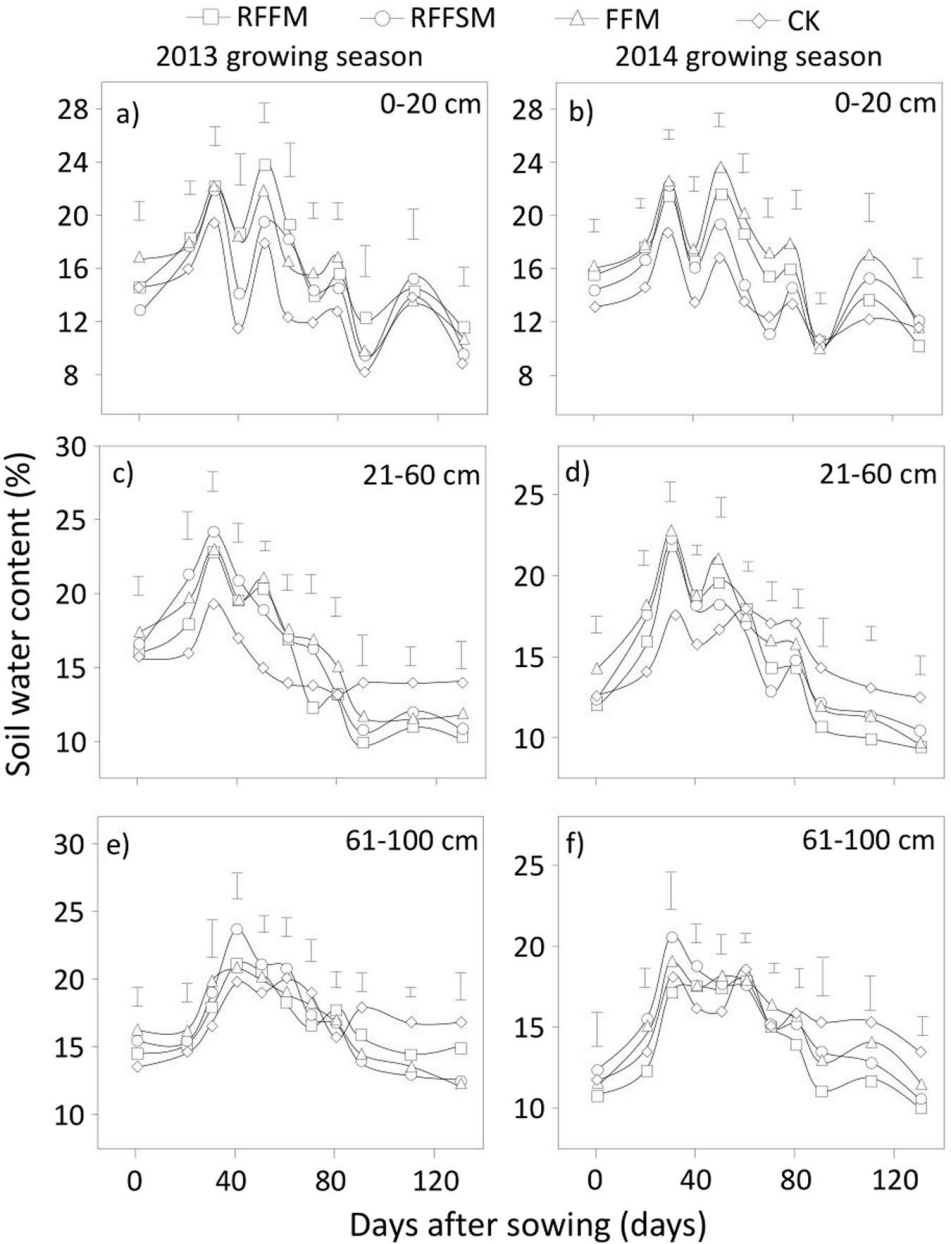
Soil water storage in different soil layers and production systems (RFFM, seedbed was prepared as alternating ridge-furrow units mulched with transparent polyethylene film; RFFSM, alternating ridge-furrow units mulched with transparent polyethylene film and wheat straw; FFM, non-contoured seedbed mulched with polyethylene film; CK, non-contoured seedbed without mulch) during two growing seasons in the Loess Plateau. Bars stand for LSD at 0.05 levels.

**Figure 2:**
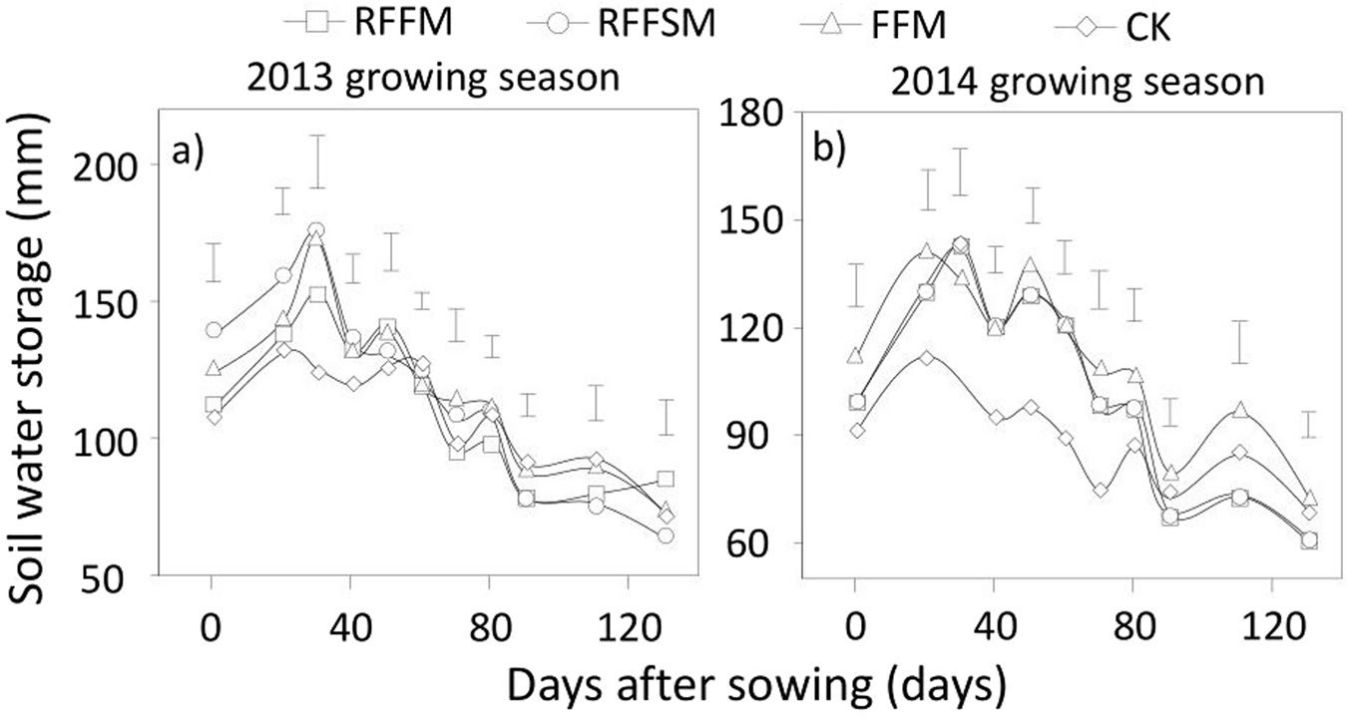
Soil water storage in 0–100 cm soil layers using different production systems (RFFM, seedbed was prepared as alternating ridge-furrow units mulched with transparent polyethylene film; RFFSM, alternating ridge-furrow units mulched with transparent polyethylene film and wheat straw; FFM, non-contoured seedbed mulched with polyethylene film; CK, non-contoured seedbed without mulch) during two growing seasons in the Loess Plateau. Bars stand for LSD at 0.05 levels.

SWS was closely associated with tillage methods, soil depth, and crop developmental stage (Fig. 1). In the 0–20 cm topsoil layer, SWS under the three film-mulched treatments was consistently higher than that of the control plots in the early growing stages, but this was not the case in the late developmental stages of maize for both experimental years, especially at maturity (Fig. 1a,b). Across the two growing seasons, moisture in the topsoil increased by an average of 9.9%, 8.7%, and 12.8% in RFFM, RFFSM and FFM, respectively, compared to CK. In the 21–60 cm subsoil layer, the three film-mulch treatments in both years showed significantly higher SWS values in the early growth stages compared to CK (Fig. 1c,d). However, during the late growth period, unmulched plots retained more soil moisture compared to the three film-mulched plots. In terms of deep soil (61–100 cm) moisture, no significant difference among treatments during the initial growth period was observed (Fig. 1e,f). However, during the later developmental stages, the amount of SWS was greater in the CK plots than the three film-mulched plots in both years. Regardless of growth stage, deep SWS in the CK averaged 5.6%, 4.7% and 7.8% higher than that under RFFM, RFFSM and FFM, respectively. Overall, the plots with film mulching retained more water (8.0% in RFFM, 13.4% in RFFSM and 14.7% in FFM) in the 0–100 cm soil layer than the CK did across the entire growing season (Fig. 2).

### Spatiotemporal dynamics of soil temperature

**Figure 3:**
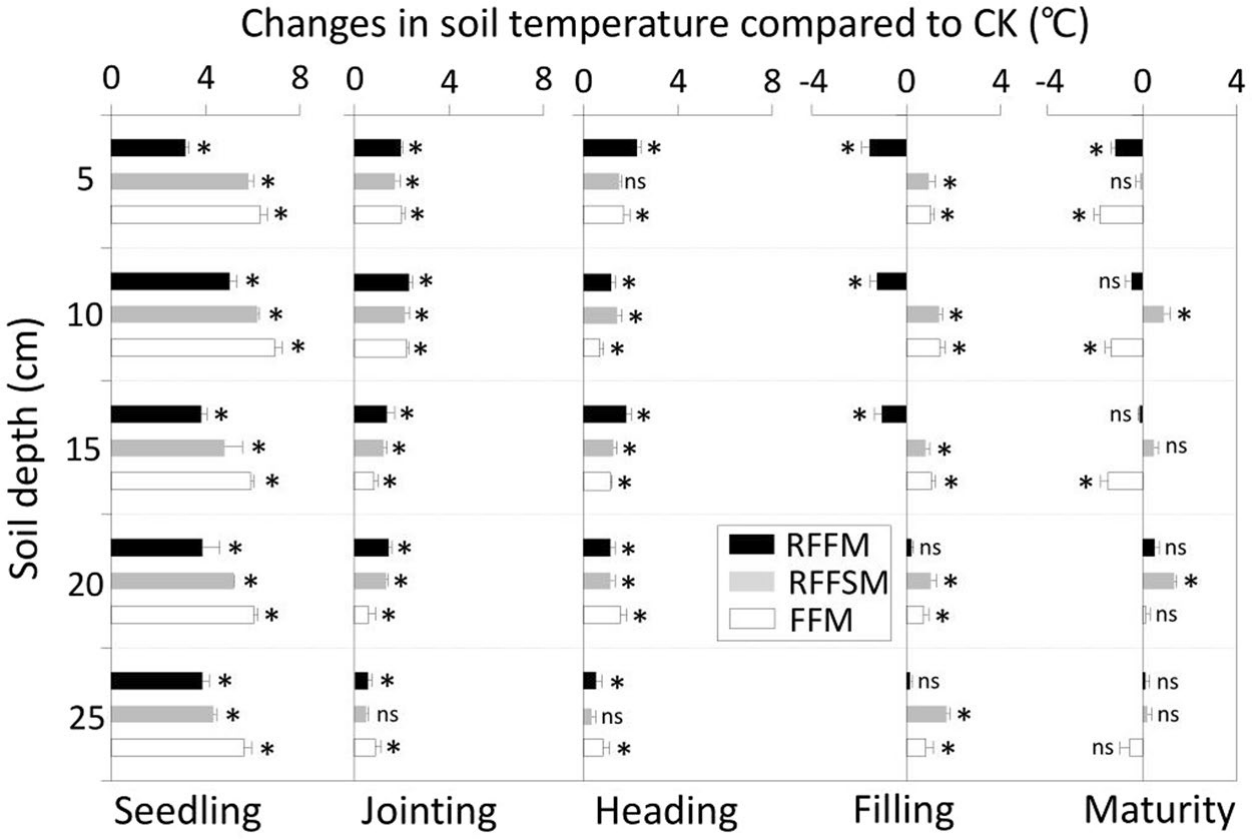
Soil temperatures in different soil layers and production systems (RFFM, seedbed was prepared as alternating ridge-furrow units mulched with transparent polyethylene film; RFFSM, alternating ridge-furrow units mulched with transparent polyethylene film and wheat straw; FFM, non-contoured seedbed mulched with polyethylene film; CK, non-contoured seedbed without mulch) during two growing seasons in the Loess Plateau. *^,^** and *** denote significance at the P < 0.05, 0.01, and 0.001 levels, respectively; ns, not significant.

Changes in soil temperatures due to film mulching were influenced by tillage methods, soil depth, and plant growth stage (Fig. 3). From seedlings to heading, a clear soil warming effect was detected in all three film-covered plots. The temperature changes were gradually reduced in each soil layer with increasing plant developmental stage. At the seedling stage, the increases in soil temperatures under the three film-mulch treatments were unaffected by soil depth. The RFFM, RFFSM and FFM treatments increased the 0–25 cm soil temperature by 3.9, 5.3 and 6.2 °C on average, respectively, compared to the CK. In both the jointing and heading stages, the warming effect was reduced with soil depth in the three film mulch plots. The 0–25 cm layer soil temperatures under RFFM, RFFSM and FFM averaged 2.0, 1.9 and 0.8 °C higher at jointing, respectively, and 1.4, 0.9 and 0.7 °C higher at heading, respectively, compared to the CK. From filling to maturing, the film-induced soil warming weakened or, in some cases, became negative. The temperature gap between the film-mulch plots and the CK decreased with soil depth. Compared to the CK, the changes in 0–25 cm soil temperatures under RFFM, RFFSM and FFM averaged −0.7, 1.2 and 1.0 °C at the filling stage, and −0.2, 0.6 and −1.0 °C, respectively, at maturity.

### Soil nutrients

**Figure 4:**
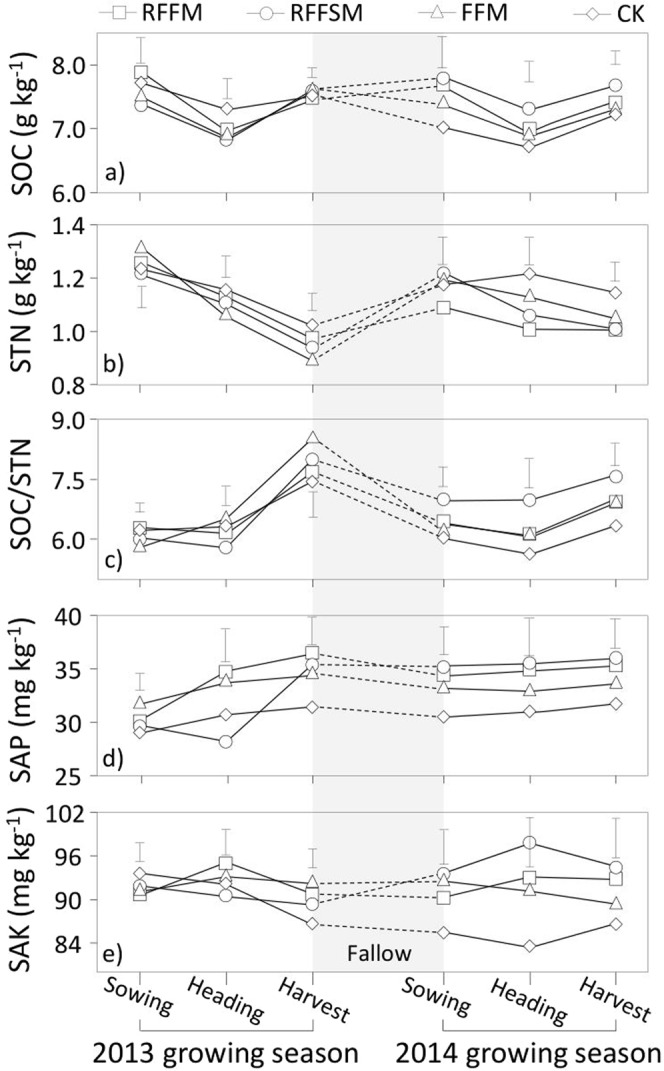
Soil nutrient parameters using different production systems (RFFM, seedbed was prepared as alternating ridge-furrow units mulched with transparent polyethylene film; RFFSM, alternating ridge-furrow units mulched with transparent polyethylene film and wheat straw; FFM, non-contoured seedbed mulched with polyethylene film; CK, non-contoured seedbed without mulch) during two growing seasons in the Loess Plateau. Bars stand for LSD at 0.05 levels.

During each growing season, SOC content in all plots tended to decrease during the vegetative period (sowing to heading) and increase during the reproductive stages (heading to maturity) (Fig. 4a). Both RFFM and RFFSM exhibited a stronger capacity for SOC recovery during the fallow season, but had greater SOC losses during the growing season. Averaged over the two seasons, SOC reduction during vegetative phase was 0.7, 1.1, 0.6 and 0.4 g kg^−1^ for RFFM, RFFSM, FFM and CK, respectively. The increasing SOC levels during reproduction were 0.6, 0.8, 0.5 and 0.4 g kg^−1^, respectively. After two growing seasons, the changes in SOC were −0.2, 0.3, −0.2 and −0.4 g kg^−1^ for RFFM, RFFSM, FFM and the CK, compared to the respective basal values.

STN levels steadily decreased during the growing season for all plots in both years, but there was a clear increase in the fallow season (Fig. 4b). The reduction of STN in the first season was not significantly different among the four treatments. However, a more pronounced decline in STN occurred in the three film-mulch plots, especially in RFFM. The average STN changes within the growing season were −0.2, −0.2, −0.3 and −0.1 g kg^−1^ for RFFM, RFFSM, FFM and CK, respectively. As a result, the C/N ratios in all treatments slightly decreased in the vegetative period but significantly increased during the reproductive stage (Fig. 4c). Compared to the unmulched CK, the three film-covered plots displayed a greater magnitude of C/N change during the vegetative and reproductive periods, especially for RFFM. Over two years, the average C/N ratios at maturity increased by 39.2%, 135.8% and 76.8% under RFFM, RFFSM and FFM, respectively, compared to the CK.

SAP content in all treatments increased with the plant developmental stage. The magnitude of this increase was greater in the first growing season than in the second season (Fig. 4d). Film-mulched treatments consistently achieved greater SAP availability during the growing season in both years. Across the two growing seasons, SAP contents in the RFFM, RFFSM and FFM treatments were 3.8, 2.8 and 2.7 g kg^−1^ higher, respectively, than in the CK. Although the SAK values under the four treatments fluctuated widely within a single growing season, the SAK contents at maturity did not significantly differ from their basal values at the sowing stage.

### Soil enzyme activities

**Figure 5:**
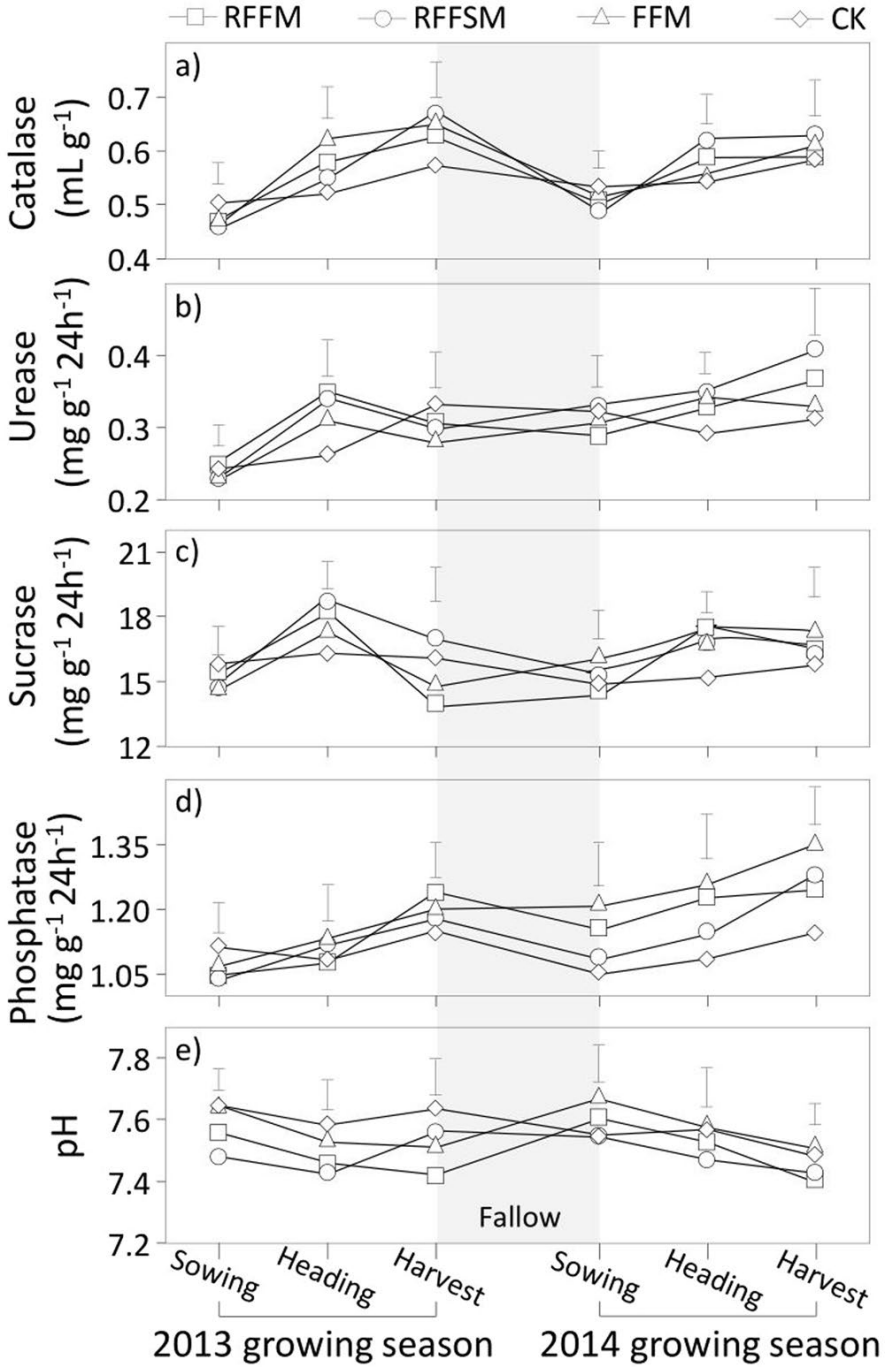
Soil enzyme activities using different production systems (RFFM, seedbed was prepared as alternating ridge-furrow units mulched with transparent polyethylene film; RFFSM, alternating ridge-furrow units mulched with transparent polyethylene film and wheat straw; FFM, non-contoured seedbed mulched with polyethylene film; CK, non-contoured seedbed without mulch) during two growing seasons in the Loess Plateau. Bars stand for LSD at 0.05 levels.

Soil enzyme activities were strongly affected by tillage methods, growth stage and farming intensity (Fig. 5). Catalase activity in the three film-mulched plots rapidly increased with plant development, especially during the vegetative period (Fig. 5a). Uncovered plots, in most cases, maintained the lowest values of catalase and displayed the slowest rates of increase. The starting (sowing stage) values for catalase did not significantly differ among treatments. At maturity, catalase activities under RFFM, RFFSM and FFM were 0.04, 0.08 and 0.08 mL g^–1^ higher, respectively, than the CK. In both seasons, urease activity increased in all plots during the vegetative period. However, the three film-covered plots had a greater increase at harvest compared to the CK (Fig. 5b). Over the two growing seasons, urease activities at the heading stage increased by 0.07, 0.07 and 0.05 mg g^–1^ 24 h^–1^ under RFFM, RFFSM and FFM, respectively, compared to the CK. Sucrase activity in uncovered plots was similar during both growing seasons (Fig. 5c). For the three film-mulch treatments, sucrase activities in each season increased during the vegetative period with greater values (26.4%, 23.5% and 16.7% higher for RFFM, RFFSM and FFM, respectively) at the heading stage compared to the CK. In contrast, film mulching led to a decrease in sucrase activities throughout the entire reproductive period. Sucrase activity in unmulched plots was relatively stable over the growing season. Phosphatase activities in all plots increased over the growing season in both years (Fig. 5d). However, film mulching was more likely to result in increased activity. Over the two years, phosphatase activities at maturity increased by 0.18, 0.16 and 0.14 mg g^−1^ 24 h^−1^ under RFFM, RFFSM and FFM, respectively, compared to their values at the sowing stage. The phosphatase increase was only 0.06 mg g^−1^ 24 h^−1^ for the CK. Soil pH values in all plots declined with crop growth, except for RFFSM during the reproductive period in 2013 and the CK at the vegetative stage in 2014. Film mulching led to a greater decrease in soil pH compared to the CK. However, the pH differences between the three film-covered treatments were not significant.

### Crop growth parameters

**Figure 6:**
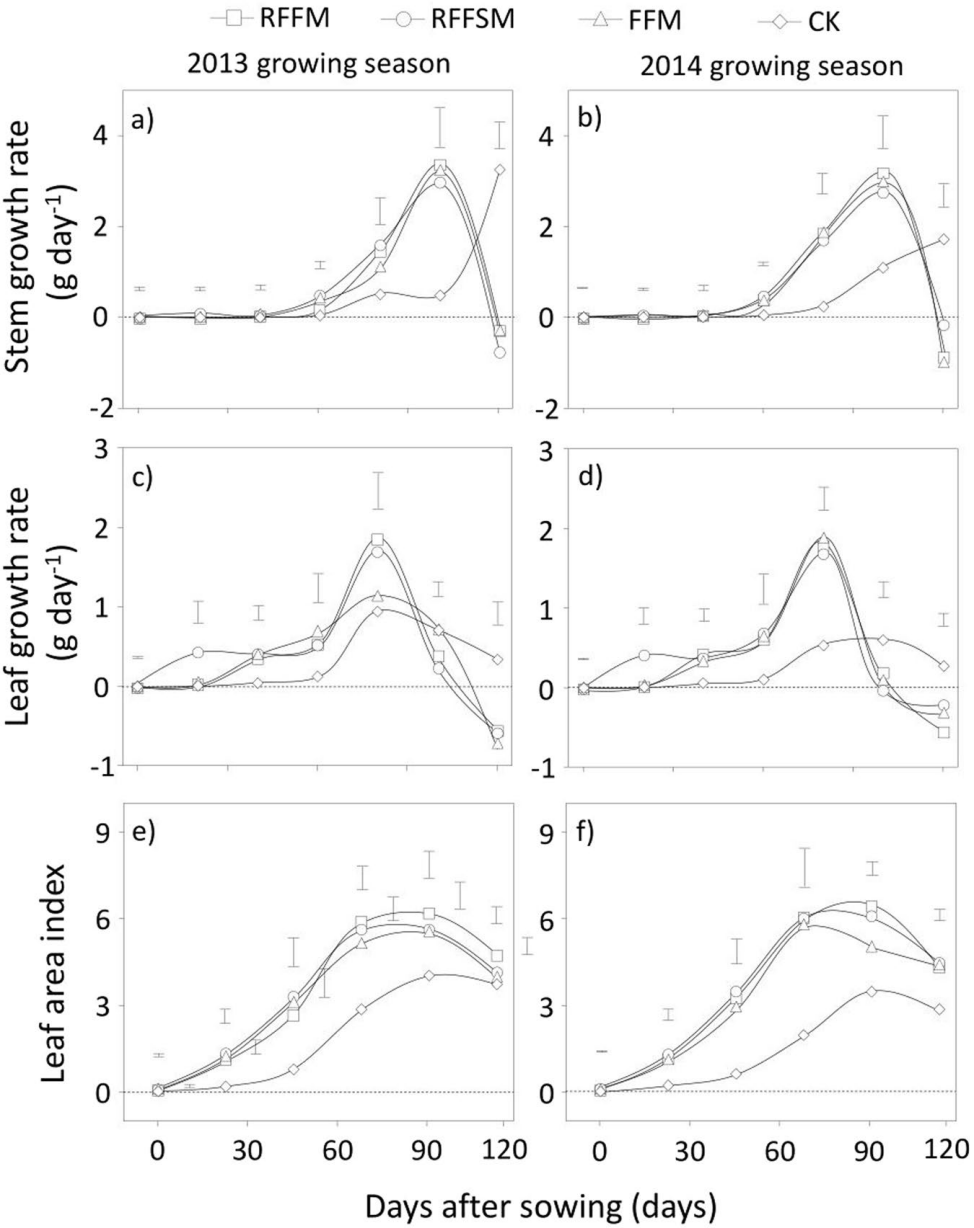
Crop growth parameters using different production systems (RFFM, seedbed was prepared as alternating ridge-furrow units mulched with transparent polyethylene film; RFFSM, alternating ridge-furrow units mulched with transparent polyethylene film and wheat straw; FFM, non-contoured seedbed mulched with polyethylene film; CK, non-contoured seedbed without mulch) during two growing seasons in the Loess Plateau. Bars stand for LSD at 0.05 levels.

**Figure 7:**
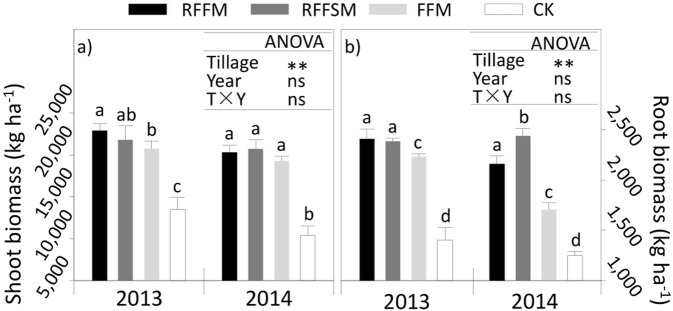
Shoot and root biomass using different production systems (RFFM, seedbed was prepared as alternating ridge-furrow units mulched with transparent polyethylene film; RFFSM, alternating ridge-furrow units mulched with transparent polyethylene film and wheat straw; FFM, non-contoured seedbed mulched with polyethylene film; CK, non-contoured seedbed without mulch) during two growing seasons in the Loess Plateau. Values followed by the same small letters within each year are not significantly different at the P < 0.05 level.

The growth rates of stems were not significantly different between treatments in the early growth stages in both years (Fig. 6a,b). However, a significant increase was observed under film-mulched plots after the jointing stage in each year. Unmulched plots displayed the lowest growth rates until the late growing season. Across the two years, the average growth rates of stems increased by 29.0%, 28.5% and 20.6% under RFFM, RFFSM and FFM, respectively, compared to the CK. Besides crop stems, the rates of leaf growth also significantly increased with film mulching in both years (Fig. 6c,d). For each treatment, the leaf growth rate had one peak value during the heading to filling stages. In both years, the three film-covered treatments had higher growth rates in most of the sample points compared to the CK. Leaf growth rates under RFFM, RFFSM and FFM were 35.0%, 50.7% and 27.2% higher, respectively, compared to the CK. As a result, LAI increased under RFFM, RFFSM, and FFM by 103.0%, 100.6% and 83.9%, respectively, over the two growing seasons compared to the CK (Fig. 6e,f). The differences in leaf/stem growth rate directly brought about changes in shoot/root biomass accumulation among the various treatments (Fig. 7). Shoot biomass was significantly affected by tillage methods but not by other factors such as Year and Year × Tillage (Fig. 7a). Similar results were observed for root biomass (Fig. 7b). Over the two years, the shoot and root biomass increased by 91.4%, 85.6%, and 72.4% and 76.9%, 85.7% and 50.2% with RFFM, RFFSM and FFM, respectively, compared to the CK.

### Yield formation and water use efficiency

Yield-related components differed in response to treatments and growth year (Table 1). The unmulched control had lower values of all yield components compared to the film-mulch plots. For each year, no significant differences in the yield component parameters among the three mulch treatments were observed, except for the abortion rate and 1,000-grain weight. The performance of most components was slightly better during the first growing season than the second season. Over the two years, the kernel abortion rates and 1,000-grain weights increased by 37.2%, 35.6%, and 22.0% and 58.7%, 47.6% and 40.1% with RFFM, RFFSM, and FFM, respectively, compared to the CK.

**Table 1:** Yield components of different production systems (RFFM, seedbed was prepared as alternating ridge-furrow units mulched with transparent polyethylene film; RFFSM, alternating ridge-furrow units mulched with transparent polyethylene film and wheat straw; FFM, non-contoured seedbed mulched with polyethylene film; CK, non-contoured seedbed without mulch) during two growing seasons in the Loess Plateau.

Year	Treatment	Abortion rate (%)	Grain number per ear	1,000-grain weight (g)	Harvest index	Yield (kg·ha^−1^)
2013	RFFM	16.2a	669.8a	34.6a	0.56a	12,863.5a
	RFFSM	14.7b	678.1a	32.7b	0.50a	11,611.5a
	FFM	18.6a	661.1a	31.6b	0.54a	11,198.2a
	CK	25.3c	495.3b	21.5c	0.41b	5,485.2b
2014	RFFM	17.6a	667.1a	31.6a	0.55a	11,175.4a
	RFFSM	20.2b	666.1a	28.9a	0.48a	10,181.1a
	FFM	23.6c	616.3b	26.9b	0.52a	9,953.9b
	CK	28.6d	412.9c	20.2c	0.39b	4,022.6c
ANOVA	Treatment (T)	**	*	**	*	*
	Year (Y)	ns	ns	ns	ns	ns
	T × Y	ns	ns	ns	ns	ns

Values followed by the same small letters within each year are not significantly different at the P < 0.05 level. *^,^**, and ***significant at p < 0.05, 0.01, and 0.001 level, respectively; ns, not significant.

Based on the improvement in yield components, the average grain yield and harvest index increased over the two years. The respective increases were 156.2% and 38.8% in RFFM, 132.4% and 22.5% in RFFSM, and 125.8% and 32.5% in FFM compared to the CK (Table 2). Both RFFM and RFFSM showed increases in WUE of 150.0% and 155.2%, respectively, compared with the CK.

**Table 2:** Grain yield and water use efficiency using different production systems (RFFM, seedbed was prepared as alternating ridge-furrow units mulched with transparent polyethylene film; RFFSM, alternating ridge–furrow units mulched with transparent polyethylene film and wheat straw; FFM, non-contoured seedbed mulched with polyethylene film; CK, non-contoured seedbed without mulch) during two growing seasons in the Loess Plateau.

Year	Treatment	Rainfall (mm)	Changes in SWC (mm)	ET (mm)	WUE (kg·ha^−1^·mm^−1^)
2013	RFFM	404.7	12.7b	417.4a	30.8a
	RFFSM	404.7	−36.3d	368.4b	31.5a
	FFM	404.7	89.9a	494.6c	22.6b
	CK	404.7	−18.8c	385.9d	14.2c
2014	RFFM	380.5	26.6b	407.1a	27.5ab
	RFFSM	380.5	−16.5c	363.9b	29.9ab
	FFM	380.5	33.1a	413.6c	24.1b
	CK	380.5	34.6a	415.1c	9.7c
ANOVA	Treatment (T)	—	**	**	***
	Year (Y)	—	*	*	ns
	T × Y	—	*	ns	ns

Values followed by the same small letters within each year are not significantly different at the P < 0.05 level. *, **, and ***significant at p < 0.05, 0.01, and 0.001 level, respectively; ns, not significant.

## Discussion

Increasing soil water availability through collecting and retaining rainfall in the soil is key to improving crop production in areas where evaporation exceeds precipitation^17^. The shape of alternating ridges and furrows can be modified to increase the efficiency of rainfall collection. The ridges reduce rainwater run-off, and the furrows aid in water conservation^18^. Rainwater channeled to the furrows penetrates deeper into the soil profile, increases SWS, and is readily available to the plants. Mulching the soil with film prevents evaporation, provides effective channeling, and improves the soil water status^19^. Consistent with the findings of previous reports, our results showed that the three film-mulch treatments significantly increased SWS in the 0–100 cm layer in both years compared to the CK. For the topsoil water (Fig. 1a,b), all three film-mulch treatments retained more water over the two growing seasons than the control plots. The increasing magnitude in water storage due to film mulch was higher than previously reported. This is probably because the rainfall amounts in both experimental years were greater than average. The water effect under film mulch should also be quantified in dry years. Besides soil water effects, mulching soil with film generally increased soil temperature and hence promoted seedling establishment and earlier development (Fig. 2). At the late growing stage, the warming effect on topsoil was not detectable due to the expanded canopy and shady soil in the mulched plots. Previous studies have examined the response of crop establishment in ridge-furrow mulch systems. Majority of the crops, especially those grown in semiarid environments, have shown a positive response^20,21^. For late plant growth stages, improved SWS could be a major driving factor for crop reproductive development and yield improvement.

Both accelerated plant growth and increased biomass accumulation due to film-mulching could intensify soil water consumption due to the larger crop canopy. This is particularly true for subsoil water storage at later growth stages^10,22,23^. One concern is that the moisture content of the growing dry layer will not be recovered until harvest due to scarce rainfall in extremely dry seasons. This could result in an unacceptable yield loss for the next crop along with greater soil degradation. In the present study, the extensively adopted ridge-furrow mulch technique (RFFM) maintained the lowest level of subsoil water storage in the late growing season in both years compared to the CK and FFM (Fig. 1c,d). This may be explained by canopy size-dependent water consumption and water collection capacity. Plants with a greater aboveground biomass often require more soil water for transpiration than smaller plants^24^. In addition, in our study area, the rainfall within the growing season is usually concentrated in the latter part of season and most precipitation occurs as heavy rains. However, the total amount of rainfall during the early growth period is always low and is composed primarily of ineffective rainfall events (less than 5 mm)^25^. The shape of the ridge-furrow incorporating full-film mulch is useful for harvesting light rainfall and preserving soil water. The film hole size permits infiltration of light rainfall, leading to less water loss through evaporation. Film-mulched ridges are effective for rainwater harvesting, even under light rainfall events and the ridges help rainwater to penetrate the soil to become accessible soil water^3^. Our results also suggest that the most extensively adopted technique (RFFM) maintained better water status during the early growth period than the CK or FFM. During the late growth stage, increased rainfall made infiltration more difficult due to the small size of the film holes. Under this situation, a large proportion of rainwater collected by the ridges may be lost. This may explain why the SWS under RFFM was significantly lower than in both CK and FFM. To resolve this issue, a novel manner of film-mulching (RFFSM) was designed, and its efficiency in water harvesting and storage was tested. We continued to use the configuration of ridges and furrows, but full-film mulching was replaced by alternating film-mulching and straw coverage. The space between the film and soil allowed more rainwater to slowly infiltrate the soil without significant evaporation. This effect may be more pronounced at later growth stages due to the heavier rainfall during this period. Based on this hypothesis, our data indicated that, compared to RFFM, SWS under RFFSM was not improved during the early season, but it significantly increased during the late growth period, particularly the subsoil. Therefore, for rainwater harvesting, RFFSM appears to be an improvement and a promising option for increasing soil water sustainability.

In addition to soil water-thermal conditions, film-induced changes in soil nutrients and soil microbial activity play important roles in maintaining nutrient availability and soil health^26,27^. Among these nutrient-related parameters, SOC is critical. The application of film mulch often accelerates decomposition of organic matter due to improved soil water–thermal status^28^. This was partially supported by our results showing that during the vegetative period, SOC under film-mulched plots was lower than SOC under the CK (Fig. 4a). This rapid decrease may be attributable to the mulched-induced improvements in soil water and temperature, which not only increased nutrient demand by more vigorous growth of crop, but also increased the activity of soil enzymes such as catalase and sucrase, leading to rapid decomposition of organic matter and a decrease in SOC content^29,30^. During the fallow season, the SOC contents increased under RFFSM and RFFM, but decreased under FFM and CK. This may be because of increased fresh organic matter input and greater root residues (Fig. 7a,b) in both treatments. The soil carbon pool can benefit from the RFFSM technique through receiving organic matter from increased crop root residue or extra carbon inputs. However, this study included field data from only two consecutive growing seasons. A positive SOC effect due to straw return was more likely to be underestimated even though the SOC level can increase synergistically with straw mulching^31,32^. For instance, at the harvest of the first growing season, no significant differences in SOC were detected among the treatments, while RFFSM had a greater SOC after two growing seasons, compared to the CK. Therefore, the study demonstrates the need for further field validation of the efficacy and stability of RFFSM in the region. Also, the determination of straw return rate should consider the local precipitation amount to meet the requirements of complete straw decomposition. In this study, the rainfall in both years was higher than the long-term mean, facilitating straw-sourced SOC input to soil. Therefore, the rate of straw return should be reduced in a drier growing season to prevent overconsumption of the straw source. Although urease activity in most stages exhibited an increasing trend of STN, soil total nitrogen in the three film-mulch plots displayed consistent reduction. One possible explanation is that the nitrogen input mainly included fertilization, crop residues, and nitrogen fixation by roots, and that these inputs did not fully compensate for the nitrogen mineralization^33^.

Under semiarid conditions, crop yield is often affected by the synchronization of plant biomass accumulation with rainwater supply during the vegetative period and the nutrient viability at the kernel formation stage^34^. Improved soil water–thermal conditions due to film-mulched ridge-furrows can accelerate early growth and increase crop biomass, eventually enhancing grain yield output and WUE^35,36^. Using the RFFM as a reference, whether the novel RFFSM technique could achieve comparable yield output and WUE remained unclear. Over the two growing seasons, the grain yield under RFFSM did not significantly differ from that of RFFM but was significantly higher than the FFM and CK treatments. The improvement in grain yield was mainly attributed to the decrease in kernel abortion and the increase in grain number and weight (Table 1). WUE was slightly higher with RFFSM than RFFM, which was due partially to the improved capacity of soil water collection in the late season (Table 2). Based on yield output and WUE, the RFFSM method is a promising farming management technique for promoting carbon storage, water retention, and boosting rain-fed maize productivity in the semiarid LP. Despite these benefits, we remain cautious regarding the use of polyethylene film in agricultural soils. Unlike biodegradable films, polyethylene sheets are mainly composed of polyvinyl chloride, which does not readily degrade in soil. In experimental studies, the film residual can be fully recovered by hand due to the use of small plot areas. When larger areas are treated with polyethylene film, mechanized film recovery needs to be developed and used to ensure sustainability of this farming practice.

## Conclusions

In semiarid areas of the LP, alternating ridge-furrow units with full-film mulching have been used since the early 2000s to improve rainwater harvesting/utilization efficiency and dryland crop productivity. Continuous cropping with this technique and long-term film mulching has reduced soil organic carbon stability and SWS sustainability. To minimize these negative impacts, we tested a novel rainwater harvesting technique that incorporated straw return into the extensively adopted ridges-furrows film mulch. SWS, especially during the rainy season, was substantially increased by this new technique. Increased root biomass residue and supplementary straw return under this system enhanced soil enzyme activity and SOC recovery. Film-induced improvements in soil water-thermal conditions via straw-associated nutrient availability directly led to better crop growth and higher yields. The grain yield and water use efficiency under this new system can be retained at high levels. Based on this study, the practice of combining straw return with film-mulched ridges-furrows is promising for dryland maize production in the semiarid Loess Plateau and it will help ensure acceptable crop yields while reducing soil degradation. However, the long-term efficacy and stability of this method will require field validation.

## Materials and Methods

### Description of the experimental site

**Figure 8:**
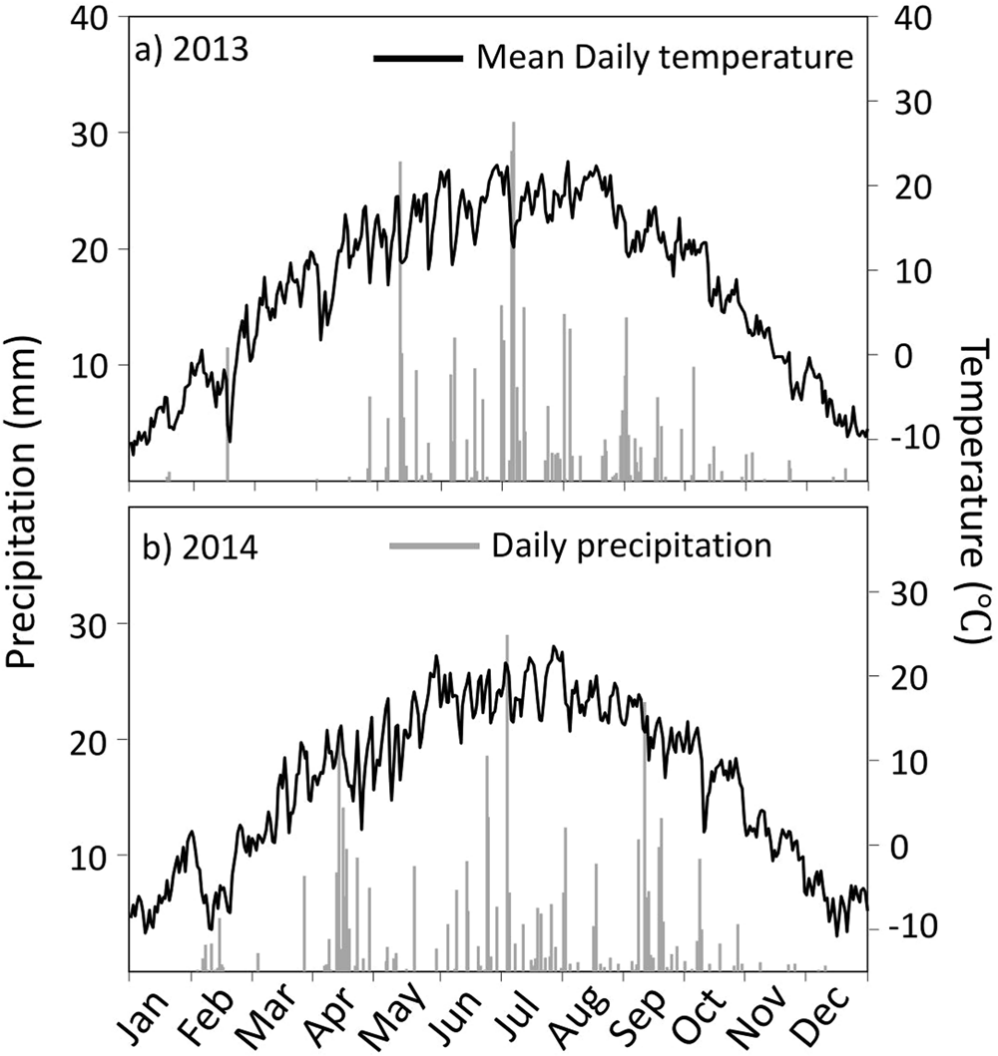
Precipitation distributions and daily mean air temperature during the 2013 and 2014 growing seasons in the Loess Plateau.

A two-year field experiment was established at the Semiarid Yuzhong Agriculture Station (35°56′34″N, 104°08′49″E), southwest Loess Plateau, over two growing seasons (2013 and 2014). The study site is characterized by a medium temperate semiarid climate. The elevation is 1970 m and mean annual temperature is 7.4 °C. The long-term average annual precipitation is 370 mm, approximately 60% of which falls during July to September. The average annual free water evaporation is approximately 1,400 mm. The rainfall during the experimental period (404.7 mm in 2013 and 380.5 in 2014) (Fig. 8) was measured using an automatic weather station (WS-STD1, England). The mean soil bulk density at 0–100 cm soil depth was 1.42 g·cm^−3^. The physical and chemical properties of the 0–30 cm soil layer were as follows: soil organic carbon, 6.8 g·kg^−1^; total soil nitrogen, 1.14 g·kg^−1^; readily available phosphorus concentration, 32.6 mg·kg^−1^; and average pH, 7.5.

### Experimental design and field management

**Figure 9:**
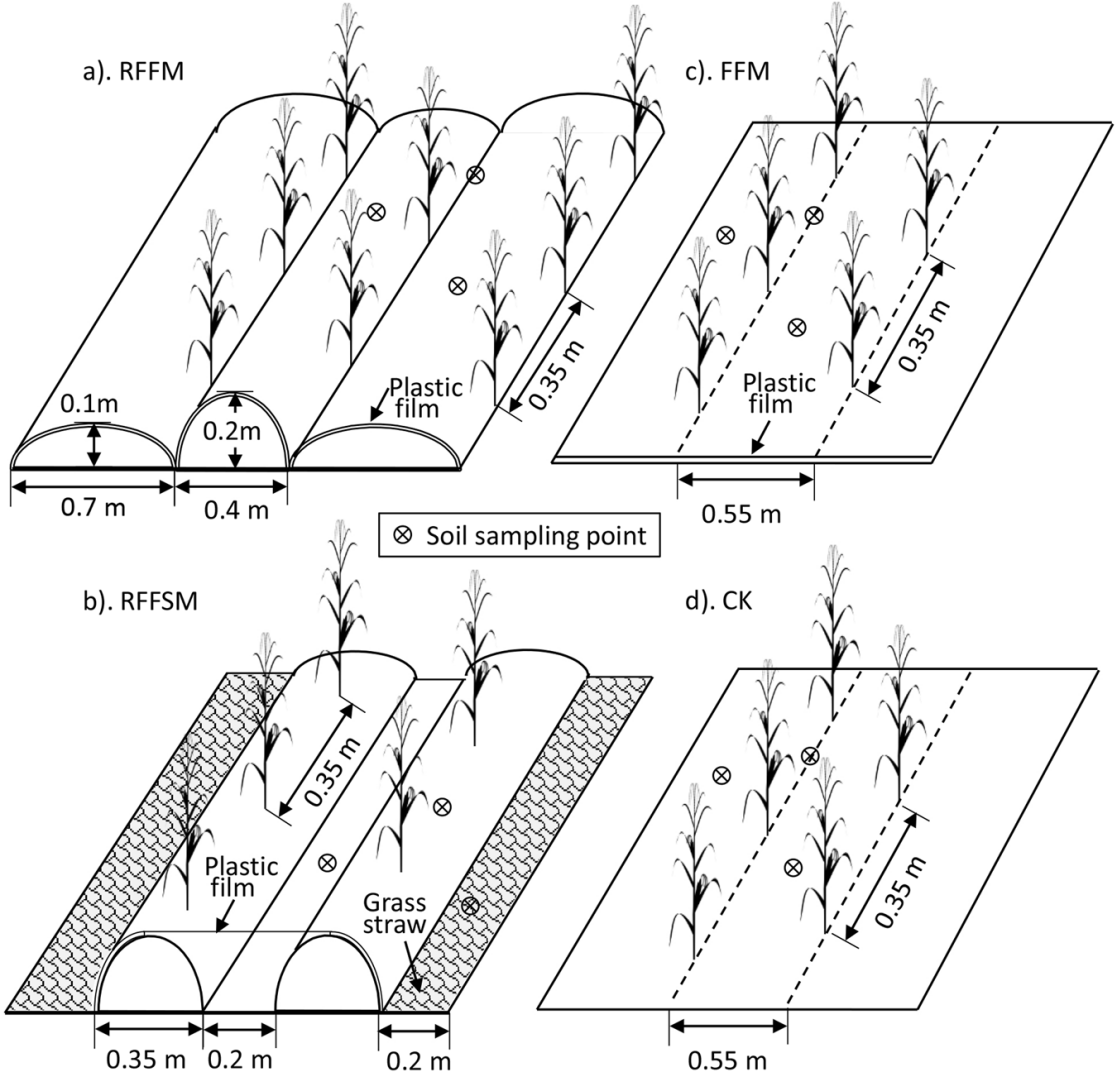
Plot layouts of various production systems used in this study.

Before sowing, a total of 12 plots were established with four plots allocated to each of the three blocks during each season. Each plot was 5.5 m long and 5 m wide. Bare ridges were made around all plots to prevent runoff. Weeds and clutter were manually cleaned from all plots. Before the ridges were made, urea (at 225 kg nitrogen·ha^−1^) and superphosphate (at 35 kg soluble phosphorus·ha^−1^) were spread on each plot. Thereafter, four different planting patterns were established: (1) RFFM, seedbed was prepared as alternating ridge–furrow units mulched with transparent polyethylene film (0.008 mm thick and 1.2 m width) (Fig. 9a); (2) RFFSM, alternating ridge-furrow units mulched with transparent polyethylene film (0.008-mm thick and 1.2-m wide) and wheat straw (Fig. 9b); (3) FFM, non-contoured seedbed mulched with polyethylene film (0.008-mm thick and 1.5-m wide) (Fig. 9c), and (4) CK, non-contoured seedbed without mulch (Fig. 9d). The treatments were arranged in a randomized complete block design with three replicates in each season. The details concerning the sizes and shapes of ridges and furrows in each treatment are presented in Fig. 9. The ridges and furrows for RFFM and RFFSM were manually set up for all prepared plots on April 20, 2013 and April 18, 2014, respectively. Once the ridges were built up, the plastic film was covered immediately for treatments RFFM, RFFSM and FFM to prevent water loss from exposed soil. The polyethylene sheet products used were all made by Lanzhou Green Garden Corporation, China. For RFFSM, air-dried wheat straw (8 cm length), obtained from surrounding fields, was covered by hand at the rate of 6 t·ha^–1^. To avoid film damage and loss of grass straw caused by late summer storms, soil was pressed on the film/straw in bands every 2 m in each mulched plot. After harvest, the plastic residue was completely removed by hand from all plots. There was a fallow period between fall harvest in 2013 and before spring planting in 2014. To reduce the labor input, the ridges set up in the first growing season remained during the fallow season for use in the next season, according to local farming practices. Before sowing during the second season, the ridges in all plots were repaired, rather than rebuilt, based on the predetermined sizes and shapes.

For each test season, a local maize cultivar, ‘*JK*-3’, was sown in the corresponding position (see Fig. 2) for each treatment using a hole-sowing machine. All plots were seeded at a population density of 50,900 plants·ha^–1^. The planting distance of each treatment was 0.35 m. Despite the difference in row spacing between RFFM and the other treatments, the number of plants in each plot was the same, allowing comparisons among treatments. The sowing dates were April 27, 2013 and April 25, 2014. During the entire growing season, the weeds in all plots were removed manually. The plants were hand-harvested on October 18, 2013 and October 10, 2014.

### Measurements and methods

Soil moisture was determined to a soil depth of 100 cm at 14-d intervals during each growing season. Before each measurement, three random sampling points located between two plants were selected in each plot. For each point, soil cores at 20 cm increments to a depth of 100 cm were sampled using an 8-cm diameter metal auger. A total of five cores were harvested from each sampling point. For each core, soil gravimetric water content (SWC%) was measured using the weighing method. Before sowing in 2013, soil bulk density at 20-cm increments within the 100-cm depth was also measured at three random points in the entire experimental field. SWS for each core was then calculated using the following equation:

$${\rm{SWS}}\,({\rm{mm}})={\rm{SWC}}( \% )\times {\rm{\rho }}{\rm{b}}({\rm{g}}\,{{\rm{cm}}}^{-3})\times {\rm{SD}}\,({\rm{mm}}),$$

where ρb is soil bulk density, and SD refers to the soil depth (200 mm).

For soil temperature measurements, five automatic temperature loggers (Micro Lite-U, Fourier, USA) were buried under the central area between two plants in each plot and were used to record hourly soil temperatures at 5-cm, 10-cm, 15-cm, 20-cm, and 25-cm depth.

### Soil nutrients

Within each plot, three soil samples at 0–30 cm depth were obtained at sowing, heading and maturity stages over the two growing seasons. To evaluate the differences of soil nutrient dynamics between treatments, each soil sample was collected by mixing the soil cores from three representative positions around each plant (Fig. 9). Each soil sample was air-dried at the time of the measurement and any visible extraneous material (e.g., residues, roots, and stones) was removed before the sample was ground, passed through a 2-mm sieve, and weighed. Soil organic carbon (SOC) and total nitrogen (STN) were measured using the dry combustion (450 °C) method with a CHNS analyzer (Elementar Vario El, Elementar Analysen Systeme GmbH, Hanau, Germany). Soil available phosphorus (SAP) was determined by extracting with 0.5 M NaHCO_3_ and analyzed using a UV-vis spectrophotometer^37^. The C/N ratio was then calculated as the ratio of SOC to STN for each sample. Soil pH was determined by a pH electrode in a 5: 1 water:soil ratio slurry.

### Soil enzyme activities

We used a titration method (0.1 mol·L^−1^ standard KMnO_4_ solution titration) to determine catalase activity, which was expressed by the number of milliliters of KMnO_4_ solution consumed by each gram of dry soil (in units of mL·g^−1^). Urease activity was estimated using the sodium phenolate method. One gram of soil was air-dried for 24 h, and the urease content was determined based on the weight of soil resident NH_3_–N. Soil phosphatase activity was measured using a disodium phosphate–benzene colorimetric assay. The mass of phenol released from one gram of soil after 24 h was used to represent phosphatase activity in units of mg·g^−1^·d^−1^. For sucrase activity determination, 5 g of air-dried soil was incubated for 24 h at 37 °C with 15 mL of 8% (weight: volume) sucrose, 5 mL of phosphate buffer at pH 5.5, and 0.1 mL of toluene. Sucrase activity was expressed by the glucose released by sucrase in units of mg·g^−1^·d^−1^.

### Crop growth parameters

Within each plot, three plants were cut at ground level at 20-d intervals over the course of the growing season in both years. Green leaf area was then measured and recorded for each plant. Leaf area was finally determined using the coefficient method according to the empirical equation:

$${\rm{Leaf}}\,{\rm{area}}={\rm{Mid}}\,{\rm{rib}}\,{\rm{length}}\,({\rm{cm}})\times {\rm{Maximum}}\,{\rm{leaf}}\,{\rm{width}}\,({\rm{cm}})\times 0.75.$$

Leaf area index (LAI) was represented by the ratio of the leaf area of each plant to the average land area occupied. Thereafter, each plant was divided into two parts (leaf and stem); these were dried in an oven at 105 °C for 1 h and then at 75 °C for a minimum of 72 h for determining their respective biomasses. The absolute growth rates for leaves and stems were calculated based on the total biomass accumulation per unit time.

### Yield components and water use efficiency

At the maize silking stage, six plants grown at the inner rows were tagged in each plot and potential kernels per ear were determined by counting the number of spikelets in each ear. The rate of kernel abortion was calculated as the ratio of aborted kernels to potential kernels per ear. During harvest (i.e., phenophase 99 according to international BBCH code^38^), the two rows of maize ears located in the middle of each plot were sampled for determination of yield components, i.e., ear length, ear diameter, kernel number per ear, kernel weight per ear and thousand-grain weight. Plot grain yield was obtained by harvesting all the ears from the middle six rows of plants. These were shelled manually and then placed into a forced-air oven at 105 °C for 1 h and at 80 °C for a minimum of 72 h.

The WUE (kg·ha^−1^·mm^−1^) was calculated using the following formula:

$${\rm{WUE}}={\rm{Y}}/{\rm{ET}},$$

where Y is grain yield (kg·ha^−1^) and ET (mm) is evapotranspiration in the crop growing season. Evapotranspiration (ET, mm) was calculated as the sum of the total rainfall during the growing season and the difference in SWS (0‒100 cm) between the beginning and the end of the growing season.

### Statistical analysis

Two-way ANOVA (PROC MIXED, SAS Institute, Cary, NC) was performed to test the effects of planting pattern and year on change of SWS, shoot/root biomass, yield performance and water use efficiency. Factors of farming pattern and year and their interactions were assigned as fixed effects while the block was taken as a random effect. Mean differences among treatments were separated by the least significant differences (LSD 0.05) test when the ANOVA showed a significant treatment effect at the P < 0.05 level. All statistical analyses were performed with SAS 9.3 software.
